# M. Edouard Dupont

**Published:** 1911-04-29

**Authors:** 


The death is announced from Cannes of M. Edouard Dupont, formerly director of the Brussels Museum of Natural History, and a well-known authority on fossils of the quaternary period. He worked at the Museum from 1863 to 1909, and was responsible for the classification of the great collections housed in the Parc Léopold. He was a Commander of the Order of Leopold, a member of the Belgian Academy, and honorary member of numerous foreign societies and academies.

